# Synchrotron soft X-ray imaging and fluorescence microscopy reveal novel features of asbestos body morphology and composition in human lung tissues

**DOI:** 10.1186/1743-8977-8-7

**Published:** 2011-02-07

**Authors:** Lorella Pascolo, Alessandra Gianoncelli, Burkhard Kaulich, Clara Rizzardi, Manuela Schneider, Cristina Bottin, Maurizio Polentarutti, Maya Kiskinova, Antonio Longoni, Mauro Melato

**Affiliations:** 1 Sincrotrone Trieste S.C.p.a., Area Science Park, Basovizza 34149, Trieste, Italy; 2 Unit of Pathology, ASS n. 2 "Isontina"Department of Anatomical Pathology, Hospital of Monfalcone, 34074 Monfalcone, Gorizia, Italy; 3 Department of Anatomical Pathology, Department of Pathology and Forensic Medicine, University of Trieste, 34127 Trieste, Italy; 4 Dipartimento Elettronica e Informazione, Politecnico di Milano, 20133 Milano, Italy; 5 IRCCS Burlo Garofolo, Trieste, Italy

## Abstract

**Background:**

Occupational or environmental exposure to asbestos fibres is associated with pleural and parenchymal lung diseases. A histopathologic hallmark of exposure to asbestos is the presence in lung parenchyma of the so-called asbestos bodies. They are the final product of biomineralization processes resulting in deposition of endogenous iron and organic matter (mainly proteins) around the inhaled asbestos fibres. For shedding light on the formation mechanisms of asbestos bodies it is of fundamental importance to characterize at the same length scales not only their structural morphology and chemical composition but also to correlate them to the possible alterations in the local composition of the surrounding tissues. Here we report the first correlative morphological and chemical characterization of untreated paraffinated histological lung tissue samples with asbestos bodies by means of soft X-ray imaging and X-Ray Fluorescence (XRF) microscopy, which reveals new features in the elemental lateral distribution.

**Results:**

The X-ray absorption and phase contrast images and the simultaneously monitored XRF maps of tissue samples have revealed the location, distribution and elemental composition of asbestos bodies and associated nanometric structures. The observed specific morphology and differences in the local Si, Fe, O and Mg content provide distinct fingerprints characteristic for the core asbestos fibre and the ferruginous body. The highest Si content is found in the asbestos fibre, while the shell and ferruginous bodies are characterized by strongly increased content of Mg, Fe and O compared to the adjacent tissue. The XRF and SEM-EDX analyses of the extracted asbestos bodies confirmed an enhanced Mg deposition in the organic asbestos coating.

**Conclusions:**

The present report demonstrates the potential of the advanced synchrotron-based X-ray imaging and microspectroscopy techniques for studying the response of the lung tissue to the presence of asbestos fibres. The new results obtained by simultaneous structural and chemical analysis of tissue specimen have provided clear evidence that Mg, in addition to Fe, is also involved in the formation mechanisms of asbestos bodies. This is the first important step to further thorough investigations that will shed light on the physiopathological role of Mg in tissue response to the asbestos toxicity.

## Background

Asbestos is the generic name of a variety of widely used in the past mineral silicates, which have been a subject of extensive epidemiological studies, since it turned out that the exposure to asbestos causes pulmonary diseases and malignant mesothelioma [1]. Asbestos fibers can enter the body by inhalation and manifest their toxicity after many years of persistence. Although since 1990 the commercialization and industrial use of asbestos have been limited and it is almost abolished today, the long latency (20-40 years) of asbestos makes the related diseases and particularly mesothelioma still an ongoing public health issue. In fact, it is predicted that the maximal number of mesothelioma cases in the world will be reached in the next ten years [2]. Northeastern Italy (provinces of Trieste and Gorizia) where massive occupational exposure to asbestos occurred in the past, is considered as one of hyperendemic for mesothelioma regions [3].

The toxicity, fibrogenicity, and carcinogenicity of asbestos have been studied for more than 50 years, but it is still unclear what are the reaction mechanisms governing the lung burden and subsequent development of fibrosis or cancer following fibre inhalation. Thus most of the aspects in the pathogenesis of asbestos-related diseases remain still undefined, preventing the development of therapeutic and prophylactic treatments.

One of the histopathological hallmarks of exposure to asbestos is the presence of asbestos bodies in the sputum or in the lung parenchyma. The asbestos body consists of an optically transparent asbestos fiber core, surrounded by a golden-brown coat containing iron-rich proteins such as ferritin and hemosiderin. The overall diameter of the body is usually from 2-5 μm and the length is typically in the range of 20-50 μm. The coating is almost never uniform and may appear segmented along the fiber into spaced spherical or rectangular units with ends that are usually knobbed.

In general, the penetration and deposition depth of inhaled fibres is determined by their length, width, shape and density. However, although fibre size and geometry are known to influence the probability of deposition and retention in the distal lung, being thus considered for carcinogenic potential [1,4], the composition and surface properties are the ones that play the major role in biological activities. The presence of transition metals in the fibres and/or their ability to absorb them is the first mechanism suggested for explaining the toxic and carcinogenic effects of asbestos. The presence of surface redox-active iron, which can be present in both ferrous (Fe^2+^) and ferric (Fe^3+^) forms, is supposed to be greatly responsible for the genotoxic and cytotoxic effects of amphibole asbestos fibres [5-10] by generating oxygen reactive species.

Among the commercially used asbestos fibres, the iron-rich crocidolite and amosite asbestos (containing 20-30% iron by weight), are considered the most carcinogenic [10-12] and are the ones most often found in lung tissues as ferruginous bodies with rare naked fibres. The presence of iron in the fibres seems to be also a key factor for the formation of the asbestos bodies *via *deposition of iron containing proteins (as ferritin). It is believed that the shell that is formed isolates the fibre from the tissue and reduces its damaging effect [11,13].

The formation of the asbestos body has been considered as a protective mechanism of the host to diminish the fiber toxicity. However, the precise mechanisms of this process have not been well defined and more important, it is not clear whether the propensity to originate asbestos bodies correlates with the established carcinogenic potential of a particular fiber type [1,11]. For example, for explaining the asbestos-mediated pathogenesis possible link between the aggregation of iron-rich proteins around the asbestos fibers and the increase of iron mediated ROS production, DNA damage and apoptosis resistance have been suggested [5,9,12].

In order to unravel the chemical and molecular mechanisms of asbestos toxicity many techniques have already been used to detect, quantify and understand the composition of asbestos bodies, but each of them has certain limitations. Optical and electron microscopies are most often used to locate and characterize the asbestos fibers [14-17]. In histological examinations, conventional optical microscopy detects the presence of ferruginous bodies but not that of the naked fibres, since most of them are too thin and their detection requires transmission or scanning electron microscopes [15]. Unfortunately, most of these microscopies require special pre-treatments of the samples that can introduce artefacts, particularly affecting the chemical investigation.

The chemical composition of asbestos bodies has mostly been investigated on digested material after extirpative procedures by different analytical techniques. A recent work, for instance, reports the use of inductively coupled mass spectrometry (ICP-MS) on extracted and multi-step digested material where among about forty-four major and trace elements associated with ferruginous bodies is even radium [18].

The composition of asbestos in histological samples has been reported for the first time recently, using particle-induced X-ray emission (PIXE) imaging [19] with a modest spatial resolution of a few micrometers.

X-ray microscopes [20], operated at the synchrotron facilities offer not only better spatial resolution but also they provide simultaneously morphological information, through absorption and phase contrast imaging, and chemical information based on X-ray Fluorescence (XRF) or X-ray absorption (XAS) microspectroscopy. The advantage of the higher penetration depth of X-rays compared to electrons has opened unique opportunities for investigating biological samples with submicrometer lateral resolution [21-24]. Here we report the first synchrotron-based X-ray microscopy (XRM) results obtained with paraffin embedded lung tissue sections containing asbestos, which evidence participation of Mg in the formation of the asbestos bodies. The asbestos bodies formed in the tissue were examined using the TwinMic instrument operated with soft X-rays (400-2200 eV) at the Elettra synchrotron laboratory [25-28]. In addition, X-ray microscopy and SEM-EDX measurements of extracted asbestos bodies were performed and compared.

## Methods

### Patients and histological sample preparation

Human samples derived from post-mortem examination of two patients with a similar history of exposure to asbestos were selected from the pathological files of the Unit of Pathology of the St. Polo Hospital of Monfalcone (Italy). Both patients were affected by pulmonary asbestosis, and one had also pleural mesothelioma. Both patients had a high content of asbestos bodies in their pulmonary parenchyma according to asbestos bodies count performed on digested lung tissue described below. For X-ray imaging and XRF analyses, 10 μm thick sections were cut from paraffin-embedded samples of non-neoplastic lung tissue, mounted on TEM gold grid (200 meshes) and air-dried. The asbestos bodies of consecutive 3 μm thick sections stained with hematoxylin and eosin according to the standard protocol were identified by a light microscope (Leica Microsystems, Germany), as shown in the Figures A1 and A2 of the additional file 1: histological examination of lung tissue of a patient with diagnosis of asbestosis.

### Asbestos body extraction

The extraction and count of the asbestos bodies were performed using a routine method [29] with some modifications. For each preparation, two samples (1-2 g) were excised from the basal side of right lung lobe, avoiding tumoral lesions, and were rapidly fixed in formalin 10%. One sample was used for asbestos extraction and the other one for calculating the wet on dry weight ratio after dehydrating procedure at 40°C for 24 hrs. For the extraction, the sample was ground, placed in 150 ml of sodium hypochlorite (20%) and left to digest at 40°C for 24 hrs. The sediment was recovered, re-suspended in 15 ml of chloroform and ethanol (50%) (1:1) and centrifuged at 800 rpm for 10 min. The supernatant liquid was aspirated, leaving about 1 ml of liquid covering the pellet that was then resuspended with ethanol and subsequently washed several times with water. The final residue was aliquoted for subsequent counting of asbestos bodies and analytical microscopy. For fiber counting, a quantified aliquot was vacuum filtered through a nitrocellulose membrane (White SM/WP 5 μm, 19 mm, Millipore, Milan, Italy). The dried filter was mounted on a glass slide and the number of asbestos bodies was counted under phase contrast optical microscope (40×). Counts are reported as number of bodies per gram of dry tissue. The counts for the two tissues in this study ranged from 50 × 10^3 ^to 500 × 10^3 ^bodies per gram (dry tissue).

### SEM-EDX analysis

The samples for SEM-EDX analyses were prepared as previously reported [13] with some modifications. Briefly, the water diluted suspension of isolated asbestos bodies was let to adhere for 60 min to glass cover-slips, followed by rapidly drying in CO_2 _ambient, and sputter-coated with gold in an Edwards S150A apparatus (Edwards High Vacuum, Crawley, West Sussex, UK). The imaging of the asbestos bodies was performed by using a Carl Zeiss XB1450 cross beam composed by an electron-beam Gelmini column allowing images at 1.1 nm spatial resolution when used at an accelerating voltage of 20 keV and at a working distance of 15-17 mm. The instrument is equipped with a SEM-EDX (FIB-Carl Zeiss) that allowed performing the chemical characterization. EDX spectra were acquired on the selected regions at a spot size of 100 nm^2 ^and 10 keV beam energy.

### Soft X-ray microscopy and XRF at TwinMic

The absorption and differential phase contrast images obtained with scanning transmission X-ray microscopy (STXM) [25,26] outline the morphological features of the specimen, whereas the simultaneous low energy XRF mapping [27,28] correlates the elemental distribution to the morphology. For the present experiments we selected X-ray energies to excite and get optimal emission conditions for elements of major interest, namely Fe, Si and Mg and other lighter elements, in particular O, relevant to the formation mechanisms of the asbestos body. The sample was raster-scanned with respect to the microprobe provided by focusing the X-ray beam using zone plate optics. Both the transmitted X-rays and the emitted XRF photons can be collected simultaneously by means of a CCD camera [26] and Silicon drift detectors optimised for low energy XRF [27,28]. The images were acquired with a spot size ranging from of 100 to 500 nm with dwell time varied between 2 s and 20 s.

The elemental distribution was obtained by processing the XRF spectra using the *PyMCA *software [30].

## Results

### SEM-EDX and XRM-XRF analyses of extracted asbestos bodies

**Figure 1:**
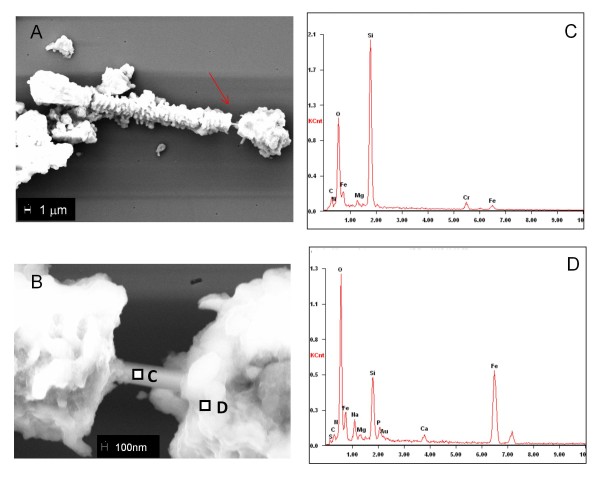
**SEM-EDX analyses of an extracted asbestos body**. Panel A: SEM image of an asbestos body placed on a glass support. In panel B higher magnification of the denudated part of the asbestos body, indicated by arrow in panel A. Panels C and D: EDX spectra acquired on the selected regions, indicated in panel B as points C and D respectively. The Cr signal originates from the glass support and should be ignored. Electron microprobe size of 100 nm^2 ^(see material and methods for instrument details).

Prior to examining the asbestos-containing tissue samples, some asbestos bodies extracted from archive tissues were analysed by SEM and XRM for the sake of comparison. Figure 1 shows representative SEM results of an asbestos body of length ~45 μm (panel A) containing a region (panel B) with almost denudated fibre (around 0.4 μm in diameter), possibly as a consequence of the extractive procedure. The EDX spectra from two 100 nm^2 ^areas of the nude fibre (panel C) and the coating (panel D) show the corresponding elemental composition. Considering the low penetration depth of the electron beam and the different densities of the analyzed regions, the EDX peak intensities should be considered only as relative. However, the EDX spectra confirm the much higher Fe content in the coating most likely as oxide, which accounts for the increased O intensity as well. From the other elements it is important to denote that comparable amounts of Mg are found both in the fibre and the coating spectra. The co-localization of Mg and Fe and the absence of Ca in the EDX spectrum from the naked fibre (panel C) suggest that it is amosite asbestos [9,14,16]. Spectra similar to that in panel C were obtained by investigating the denudated internal core of most of the analysed asbestos bodies after different preparations: they showed similar spectra, but some of them, as previously reported [13], showed also the presence of crocidolite. The EDX spectra from the coating (panel D) show the presence of elements, mainly resulting from endogenous tissue reactions. As expected, the relative peak heights confirm the much higher Fe content in the coating with respect to the naked fibre. In the coating also some Ca and Na are present, whereas Si should be contribution from the fibre and/or the glass support.

**Figure 2:**
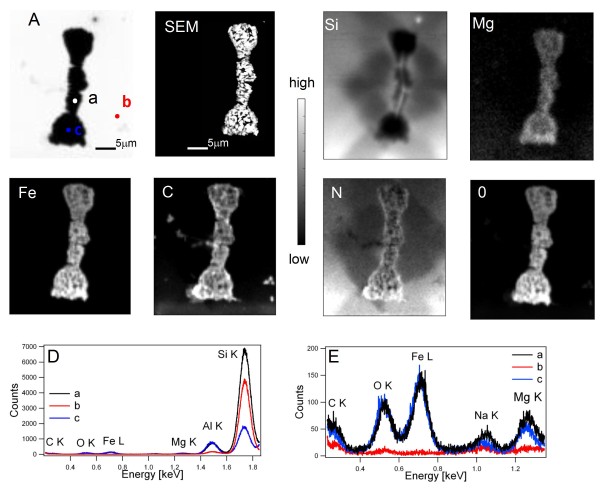
**Soft X-ray microscopy and XRF analyses on an extracted asbestos body**. The images show the analyses of an extracted asbestos body deposited on a Si_3_N_4 _window. Panel A: X-ray absorption image acquired at 1.935 keV (40× 48 μm^2 ^area, 20 ms dwell time, 500 nm spot size). Panel SEM: SEM image of the same asbestos body. Panels Mg, Si, Fe, N, O and C: XRF maps acquired at 1.935 keV with 6 s/pixel dwell time (Si and Mg) and at 0.9 keV (Fe, N and O) with 4 s/pixel dwell time, both with 500 nm spot size. Panel D and E: XRF spectra collected on the points indicated in panel A at 2 keV and 1.4 keV respectively (30 s acquisition time).

The XRM-XRF results of the extracted asbestos bodies in Figure 2 are in general agreement with SEM-EDX analyses but apparently the XRF analysis provides more complete quantitative chemical information, since it can reveal the presence of buried matter, thanks to the much higher probing depth of the X-rays compared to electrons. Another very important advantage demonstrated by the results in Figure 2 is that the lateral chemical composition can be directly correlated to the morphology of the asbestos body. The absorption image (panel A) shows the strongly absorbing structure of the asbestos body, where the darkest regions clearly identify the extremes at the top, bottom and central part. The corresponding XRF elemental maps, where the brighter parts indicate the highest elemental concentration, reveal the lateral distribution of the elements under consideration. The Si XRF map clearly evidences the location of the asbestos fibre buried inside the body, confirmed by the XRF spectra taken in the selected central spots, a and c. One can see that the highest brightness in the Si map is concentrated only inside the central part of the body outlining the fibre appearance. Note that the high brightness in the Si and N maps outside the body is due to the XRF emission from the Si_3_N_4 _window supporting the specimen. The Si_3_N_4 _window emission is strongly suppressed within the dense body region, where only the emission from the thin asbestos fibre inside is clearly seen. The other XRF elemental maps indicate similar lateral distribution of Mg, Fe, N and O.

It should be noticed that the aggregation of organic matter containing Fe and Mg around the fibre is more pronounced at the two ends of the fibre, whereas the material along the fibre walls is relatively thinner. An increased density and a larger material deposition at the extremities is a frequent characteristic of the asbestos bodies, possibly related to the increased reactivity and damaging effect of the fibre tips [31]. Close inspection of the Mg and Fe maps also reveals very small differences, in particular the central part seems a bit thinner in the Mg map. The C and N maps are indicators of the presence of organic matter, whereas the O map is a mixed contribution from the organic matter and Fe, Mg and other elements that are usually present as oxygen containing compounds. These results are in agreement with previously reported high presence of proteins and other organic materials in the coating of asbestos bodies [11,13]. In accordance with SEM-EDX analyses, some presence of Na in the extracted bodies is also detected in the XRF spectra (panel E). The Na maps, reported in supporting information (additional file 2), show uniform Na distribution.

### Soft X-ray microscopy and XRF of human lung tissue containing asbestos

**Figure 3:**
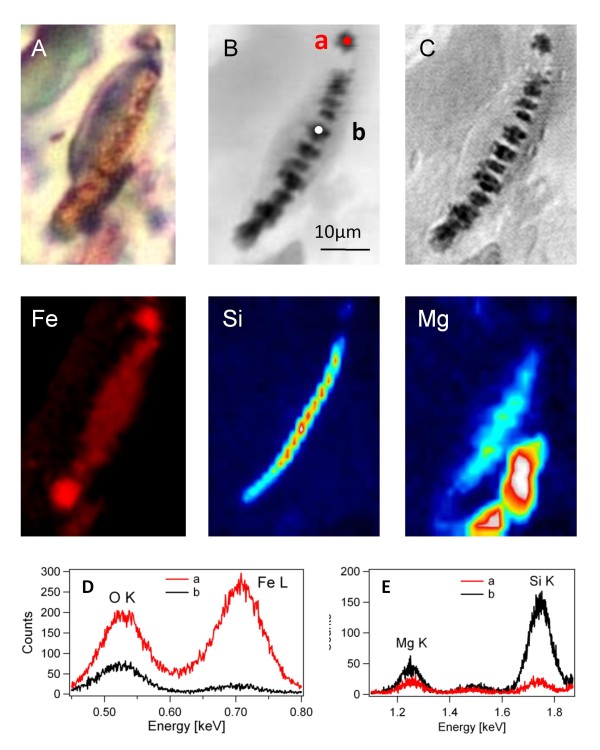
**Soft X-ray microscopy and XRF analyses of an asbestos body in human lung tissue**. Panel A: visible light image of the tissue with the asbestos body. Panels B and C: absorption and phase contrast images respectively acquired at a photon energy of 2018 eV (25 × 40 μm^2 ^size, 30 ms dwell time, 415 nm spot size). Panels Fe, Si and Mg: elemental distribution (25 × 35 μm^2 ^size, 30 s/pixel dwell time, 1 μm spot size) among the area of panels B and C, acquired at 2 keV, (Si and Mg), and at 1,3 keV (Fe). Panel D and E: XRF spectra collected in the points a and b (see panel B) at 1.3 keV (D) and at 2 keV (E) with 180 s acquisition time).

Figure 3 shows simultaneously acquired transmission X-ray images, outlining the morphology of a histological tissue with a strongly absorbing asbestos body, and the corresponding XRF maps. The X-ray images provide much more detailed morphology information compared to the optical microscope image (panel A). The absorption contrast (panel B) clearly reveals the presence of very strongly absorbing periodically assembled segments of ~1-2 μm^2^, the edges of which are well defined in the phase contrast map (panel C). The segments appear separated along the fibre and surrounded by a dense, but less absorbing organic matter. The Si map in Figure 3 outlines the asbestos fibre located inside the segmented area, with intensity variations along the fibre. Close inspection of the variations in the Si content, reveals that there is not full correspondence between the darkest areas in the absorption maps and the highest Si signal in the Si map. This is confirmed by the XRF spectra in Figure 3, panel D and E, taken in the selected spots (a and b). They clearly show that Si is almost absent at the two ends of the body, where the Fe content appears very high. This observation is in fair agreement with the results in Figure 2, obtained for the isolated body. This also suggests that apparently, there is no direct correlation between the dimensions and shape of the deposited coating and those of the original asbestos fibre.

The Mg map shows significant aggregation of this element around the asbestos fibre. Interestingly, the highest Mg content is present in some tissue areas in close vicinity of the body, which apparently accounts for the corresponding darker areas, visible in panels B and C.

The Fe map shows that the high content of this element is at the two edges of the body appearing dark in the transmission image, since Fe is strongly absorbing. On the other hand the Mg concentration at the two edges is lower, as confirmed by the microspot spectra in panels D and E, which highlight the quantitative differences between the central and edge parts of the body. In addition, Fe appears not present in the Mg-rich tissue areas in the closest proximity of the body. The Na distribution appears rather featureless, not showing specific aggregation in any structure of the imaged area. (Figure 3 in supporting information file 2).

**Figure 4:**
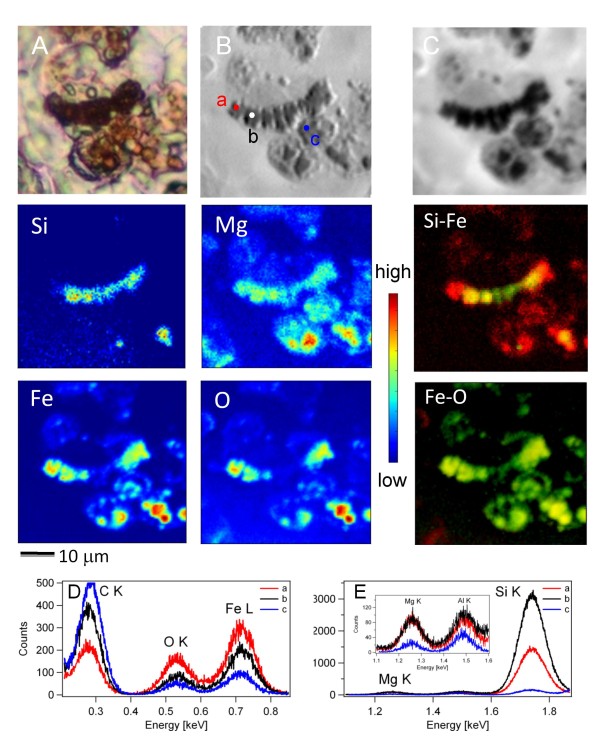
**Soft X-ray microscopy and XRF analyses of asbestos and ferruginous bodies in human lung tissue**. Panel A: visible light image of the analysed asbestos body. Panel B and C: phase contrast (B) and absorption (C) images respectively acquired at photon energy of 1.3 keV (50 × 50 μm^2 ^size, 10 ms dwell time, 250 nm spot size). Other panels (50 × 50 μm^2^, 7 s/pixel dwell time, 500 nm spot size): XRF maps acquired on the region showed in panel C, at 1.3 keV (Fe and O) and at 1.93 keV (Mg and Si). Panel Si-Fe: colocalization of Si (in green) and Fe (in red). Panel Fe-O: colocali**z**ation of Fe (in green) and O (in red). Panel D and E: XRF spectra acquired in points a, b and c (see panel B) at 1.3 keV (D) and 2 keV (E) with 175 s acquisition time.

Figure 4 shows another set of data for a tissue section containing a larger number of ferruginous bodies of different shapes, as verified by the optical microscope (panel A). The morphology of the asbestos body together with other ferruginous formations not clearly connected to fibre-like structures are seen in the absorption and differential phase contrast images (panels B and C). The edge enhancement of the phase contrast image (panel B) allows identifying inside the asbestos body the presence of a fibre with apparent diameter of about 1 μm, connecting the strongly adsorbing segments. The images also contain some other very strongly absorbing regions with distinct morphology and boundaries and other nano and micrometric formations of variable absorbing power, indicative of a different local density and/or chemical content. Comparing the Si, Mg, Fe and O XRF maps one can immediately see that almost all features in the Fe, Mg and O maps can be correlated to the segments and other stronger absorbing structures in the transmission images. The co-localization of O, Mg and Fe also indicates that both Mg and Fe are present as O-containing compounds. The Si image evidences better the location and the appearance of the asbestos fibre inside the asbestos body and some less specific particulates. It outlines the irregular shape of the fibre and other fibre fragments or particulates present in some 'dark' regions of the transmission image. Both Mg and Fe distributions can be correlated to Si, but again there are distinct areas in the vicinity of the asbestos body with high Mg content where no Si is present. Closer inspection of the Si, Fe and Mg maps also reveals that in the regions where the three elements are colocalized, Mg and Fe signal levels are more uniform indicating that these two elements are more abundant in the coating of the asbestos fibre. It appears that Mg and Fe are present in all the red-brown nodules of the tissue section (see optical image), with maximal signal in some strongly absorbing regions. However the signal of these two elements is reduced in the central part of the asbestos body where the strong absorbing dense material is present (Figure 4, panel B). This suggests the presence of some masking absorbing material in the central part of the fibre, containing other heavier elements or other dense formations that attenuate mainly the lower energy Fe and O (705 eV and 525 eV) emission, compared to the more energetic Si one (1740 eV). Comparing the Mg and Fe maps again the relative local Mg and Fe content in the well defined body in the centre of the image does not change in concert: the highest Fe concentration is at the edges, beyond the Si fibre, whereas the Mg concentration appears a bit lower. The Na map (additional file 3) does not indicate an enhanced presence of this element in the asbestos body. Very similar results were obtained examining other two tissue samples from other two patients (data not shown).

## Discussion

As noted above, most of the studies consider the fibre coating as a result of mineralization mechanisms that involve proteins as ferritin and albumin [11,13], organic acids as oxalate [32], and recently the presence of about forty chemical elements has been supposed to be involved [18]. These conclusions were based on *in vitro *experiments, mainly analyzing the composition of the asbestos body after digestive extraction from lung tissue specimens. Our X-ray microscopy results compared to the SEM-EDX analyses of extracted bodies clearly provide additional morphological and chemical information confirming the active participation of Mg in the formation mechanisms. In particular, they showed rather uniform Mg distribution in the asbestos bodies, not limited to the internal core as expected for Mg containing asbestos fibres (Figure 1, panel A).

However, since the analysed samples were isolated from the tissue by acidic and moderately aggressive chemical procedures, the synchrotron XRF and SEM-EDX results might be affected by possible chemical modifications and element loss and/or addition. For this reason, less ambiguous conclusions can be drawn from the results obtained for tissues containing asbestos bodies in Figures 3 and 4, which is the best approach to shed light on the formation mechanisms, excluding the possible errors that can be introduced by the body extraction procedure.

The potential of X-ray imaging and elemental mapping has allowed unambiguous recognition of the constituents in the asbestos bodies with submicrometric resolution and may be considered as a new methodological approach to advance histological investigations. The correlation between the morphology and lateral distribution of Si, Fe and Mg has evidenced the trend of the organic matter containing more strongly absorbing elements, (Fe, Mg, etc) to surround and isolate the asbestos fibre. Additional important information concerns the weaker absorbing organic matter: homogeneous accumulation of additional organic material surrounding the asbestos body is clearly evidenced in the X-ray images (particularly in Figure 3, panels B and C). Another finding is that the Mg and Fe spatial distribution and the appearance of the body in the tissue are not fully identical to those obtained for the extracted asbestos bodies (Figures 1 and 2). Although quantitative analyses were not performed, the presence of these elements in the asbestos bodies appears more abundant in tissue sections.

A very interesting feature in analyzing the tissues is that the Fe and Mg accumulation is not only correlated to the location of the fibre (Si), but an increased content of these two elements, in particular Mg, can be found in tissue areas in the vicinity of the body (Figures 4). In fact, the Fe and Mg presence in the 'unspecific ferruginous bodies' may appear as a lateral spreading of the inflammatory events [33]. Indeed, the most intriguing result of the present study is that Mg is found not only within asbestos bodies, co-localized mainly with Fe and O, but it also appears accumulated alone in some regions of the lung tissue in proximity of the asbestos and other ferruginous bodies. This result suggests that Mg should be considered as an active participant in the biochemical process building the coating to isolate the asbestos fibres from the surrounding tissue, and not as a constituent of the asbestos fibres, as proposed in [19].

Another mechanism suggested by *in vitro *experiments is that the asbestos fibres may release Mg and Fe in the medium with percentages that vary according with fibre composition and particularly when in contact with animal cells [34]. The sole contribution of this mechanism to explain Mg distribution in tissues around asbestos is also ruled out by our results, since the XRF Mg and Fe maps in Figures 3 and 4 clearly show that the concentration of Fe and Mg is much higher in the parts outside the fibre core. While Fe clearly should derive from iron-storage proteins such as ferritin and hemosiderin, concurrent mechanisms of mineralization and different material stratifications could be proposed for explaining the Mg aggregation.

Mg is a microelement with many physiological functions, participating in numerous enzymatic reactions. It has been demonstrated that an enhanced Mg content is a feature of inflammatory tissues and certain cancer diseases [35,36]. Other experimental findings showed that Mg modulates cellular events involved in inflammation and tissue repair [37,38]. The high presence of lymphocytes and macrophages in the areas surrounding the analyzed asbestos bodies (Figures A1 and A2 of the additional file 1) could indicate that Mg presence is linked to inflammation responses.

It was reported that Mg is involved in the visceral calcification associated with the metastatic calcification metabolic disorder [39], and it was suggested that the presence of Mg in calcifications (whitlockite) increases the stability and persistence of calcium precipitates compared to those in calciphylaxis (calcifications without Mg), with concomitant reduced inflammatory reactions. From these concepts, it could be speculated that the presence of Mg in asbestos coating is a result of some peculiar calcification mechanisms that may be the reason of the insolubility and the long persistence of asbestos bodies [31,33].

Another recent concept is that Mg participates in many mechanisms of the antioxidant defences of the body [40,41]. Although the entire picture has not been understood yet, one can suppose that its presence in regions with high iron content contributes to counteract the oxidative stress reactions that may be evoked.

Our present investigations cannot disclose the mechanisms resulting in Mg aggregation around asbestos fibres and the reason for the laterally enlarged accumulation in the surrounding tissue areas, but they strongly point to a new mechanisms to be in-depth investigated and that could have important physico-pathological implications. The possible participation of other chemical elements (particularly Ca and heavier metals) remains to be elucidated by complementary techniques, particularly higher energy XRF microscopy.

## Conclusions

The present results on Mg participation to tissue reaction to asbestos fibres in lung and the first use of synchrotron X-ray microscopies for chemical and morphological analyses in this field open a route to further in-depth investigations on the molecular mechanism of asbestos toxicity. Due to the central role of Mg in many cell and tissue mechanisms, particularly oxidative stress defence and inflammatory conditions, significant future discernment is expectable from our observations. An increased number of analyses with the presented techniques, using samples from patients with different exposure history, is also claimed in order to shed light on the role of fibre composition, shape and structure. The involvement of specific Mg-triggered molecular mechanisms, as well as the participation of other chemical elements, remains to be elucidated by complementary techniques.

Finally, it is also worth noting that some engineered nanomaterials considered also for biomedical applications, e.g. C nanotubes, have morphology, bio-durability and persistent presence in the human organs resembling those of the asbestos fibres [42-45]. Thus addressing and understanding the mechanisms of the asbestos toxicity will provide general knowledge about potential health hazards triggered by nanoparticles with similar properties.

## Competing interests

The authors declare that they have no competing interests.

## Authors' contributions

LP designed and coordinated the study, was substantially involved in acquisition, analysis and interpretation of results, and drafted the manuscript. AG was substantially involved in data acquisition at TwinMic and analyses and revised the manuscript regarding the methodological aspects. BK supervised data acquisition and analyses at TwinMic revising critically the manuscript. CR was substantially involved in designing and coordinating all the clinical aspects of the study, she revised the manuscript with clinical interpretation of the results. MS was involved in human sample selection and diagnosis, revising also the manuscript. CB contributed in the technical aspects of treatment of human samples and was responsible for asbestos body isolation and counting. MP provided SEM-EDX analyses and interpretation, contributing in revising the manuscript. MK actively contributed to data interpretation and critical and thorough revision of the manuscript. AL provided his expertise in XRF detection systems supervising data analyses and custom developed software. MM directed the work providing, as expert in diagnosis of asbestos-related diseases, the rational interpretation of the study, he revised critically the manuscript. All authors have read and approved the final manuscript.

## Supplementary Material

Additional file 1 **Histological examination of human lung tissue**. Figure A1 and A2 are microphotographs from the histological sections used for the study, colored in hematoxilin and eosin. For the diagnosis of asbestosis we have to see in lung tissue a diffuse interstitial fibrosis away from tumor zones or other lesions, associated with asbestos bodies. In Figure A1 diffuse fibrosis, ferruginous bodies and asbestos bodies are evident. There were sparse leucocytes and macrophages. In Figure A2 at higher magnification two asbestos bodies (arrows) are visible in a background of fibrosis. Click here for file

Additional file 2 **Supporting information for Figure**2**and Figure **3. Figures display absorption and XRF Na distribution of the extracted asbestos body of Figure 2, and a comparison of Si, Mg and Na elemental maps of the tissue section of Figure 3. See Figure 2 and 3 legends for technical details. Click here for file

Additional file 3 **Supporting information for Figure **4. Figures show phase contrast and Na distribution (XRF map) related to Figure 4. Spectra depict the low intensity signal from Na, compared to Mg (see XRF spectrum). See Figure 4 legend for technical details. Click here for file
