# Supplementary material for: Synchrotron soft X-ray imaging and fluorescence microscopy reveal novel features of asbestos body morphology and composition in human lung tissues
Source: Part Fibre Toxicol. 2011 Feb 7;8:7. doi: 10.1186/1743-8977-8-7 (PMC3041679; doi:10.1186/1743-8977-8-7)
Supplement: Additional file 1 — Histological examination of human lung tissue. Figure A1 and A2 are microphotographs from the histological sections used for the study, colored in hematoxilin and eosin. For the diagnosis of asbestosis we have to see in lung tissue a diffuse interstitial fibrosis away from tumor zones or other lesions, associated with asbestos bodies. In Figure A1 diffuse fibrosis, ferruginous bodies and asbestos bodies are evident. There were sparse leucocytes and macrophages. In Figure A2 at higher magnification two asbestos bodies (arrows) are visible in a background of fibrosis. [file 1743-8977-8-7-S1.PDF]

Histological examination of human lung tissue

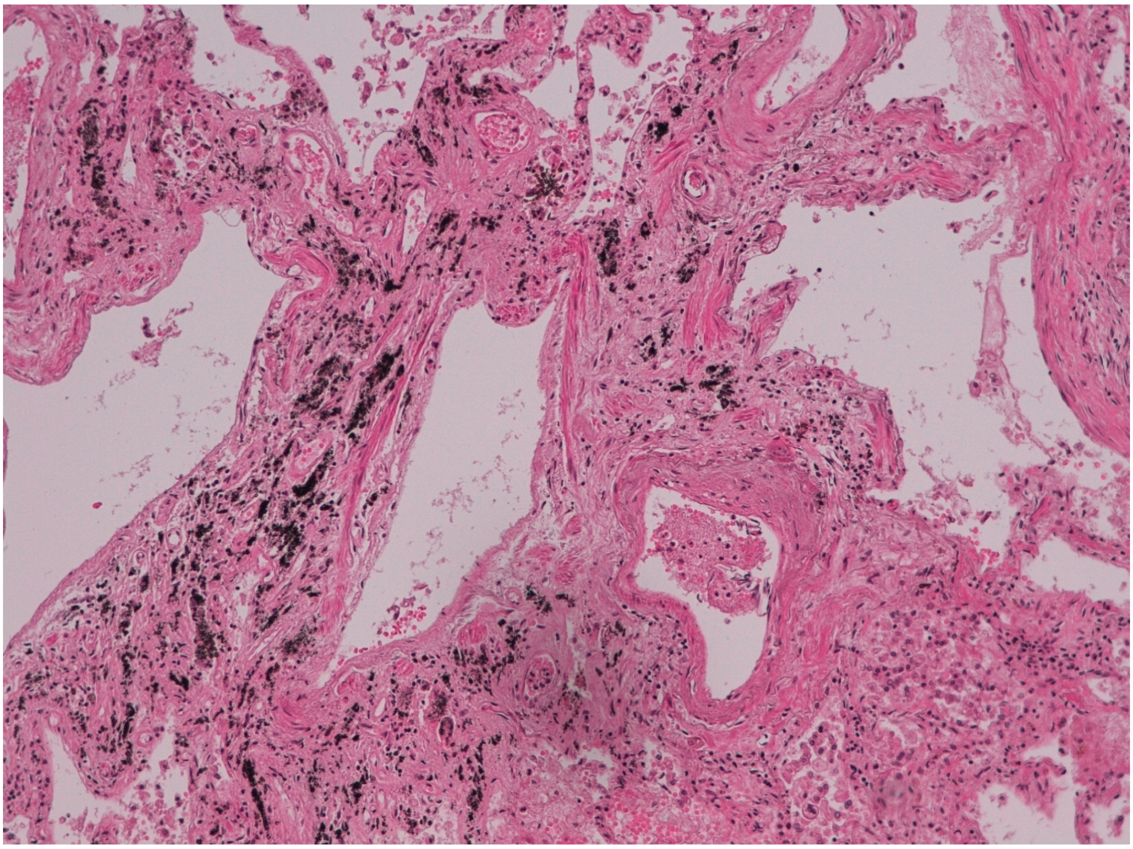

Figure A1: Tissue section.

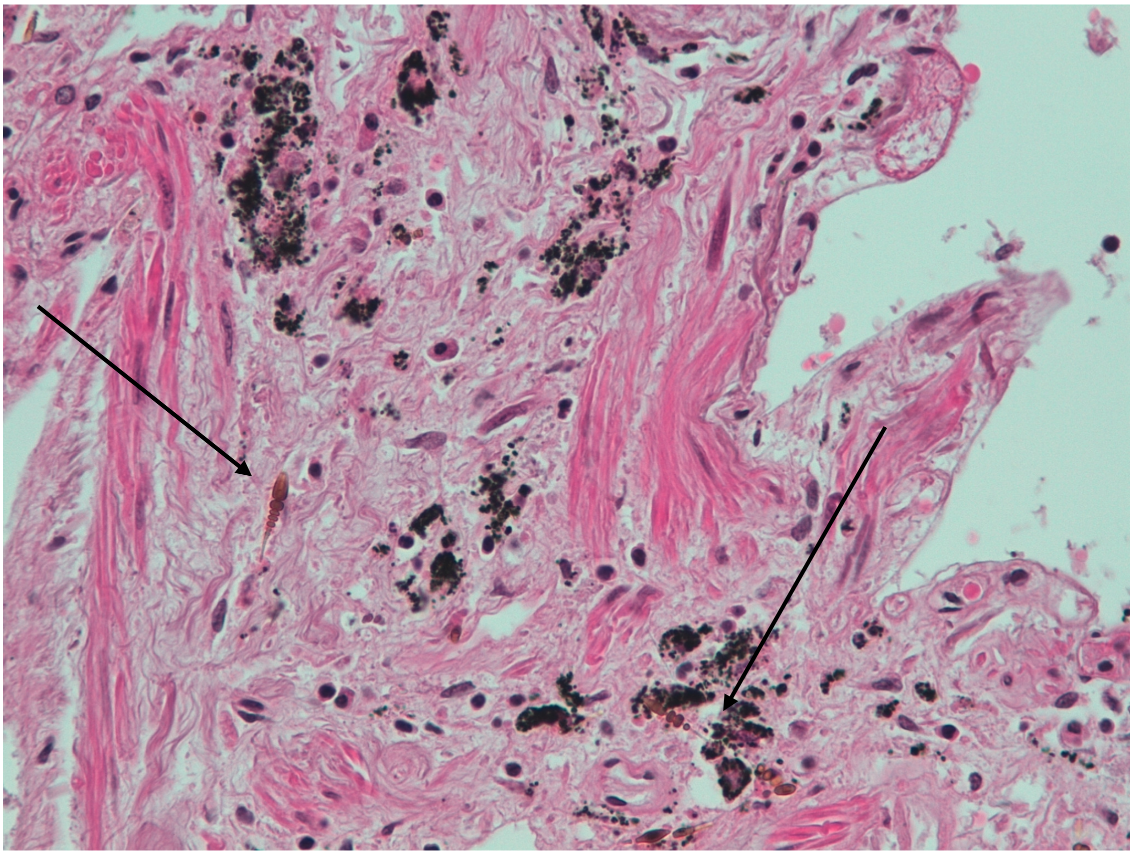

Figure A2. Tissue section at higher magnification; arrows indicate asbestos bodies.
