# Supplementary figures and images for: Synchrotron soft X-ray imaging and fluorescence microscopy reveal novel features of asbestos body morphology and composition in human lung tissues
Source: Part Fibre Toxicol. 2011 Feb 7;8:7. doi: 10.1186/1743-8977-8-7 (PMC3041679; doi:10.1186/1743-8977-8-7)

### Supplement information for Figure 2

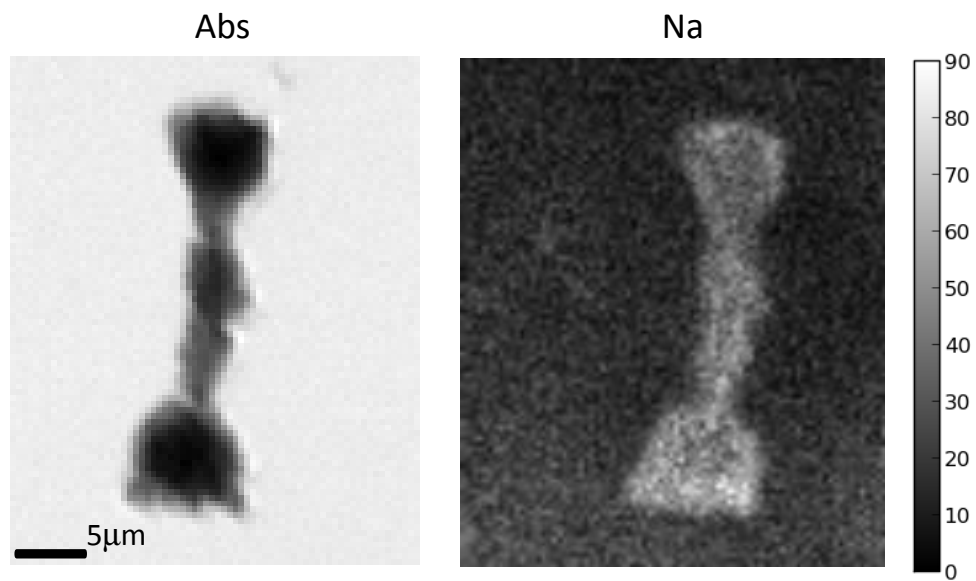

### Supplement information for Figure 3

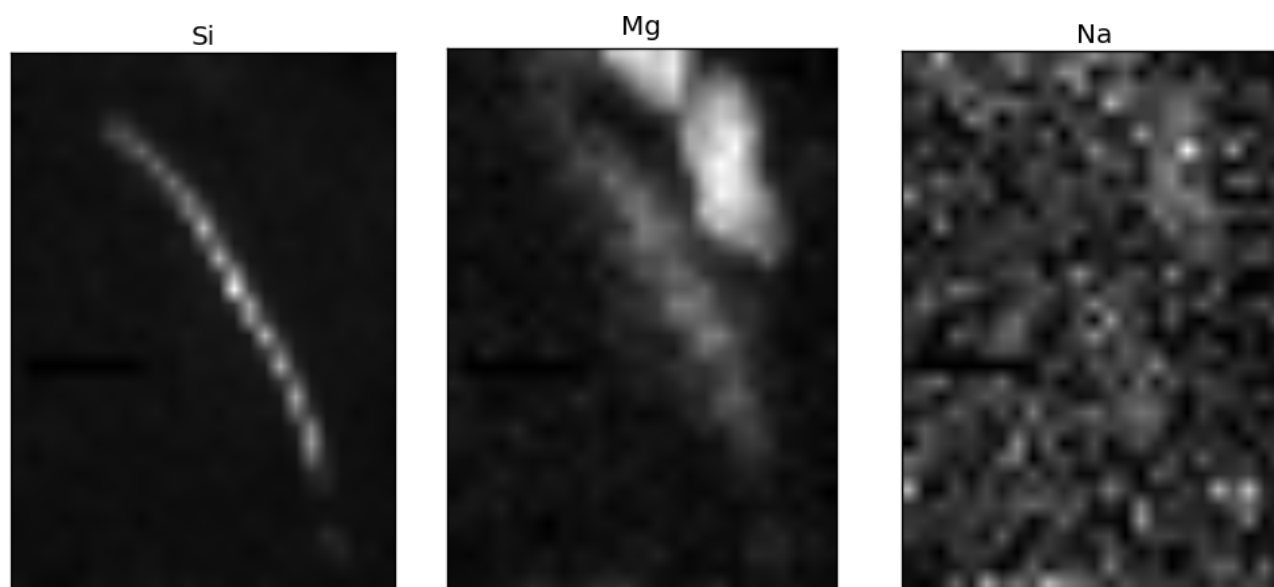

Supplement: Additional file 2 — Supporting information for Figure2and Figure 3. Figures display absorption and XRF Na distribution of the extracted asbestos body of Figure 2, and a comparison of Si, Mg and Na elemental maps of the tissue section of Figure 3. See Figure 2 and 3 legends for technical details. [file 1743-8977-8-7-S2.PDF]

## Supplement information for Figure 4

Abs

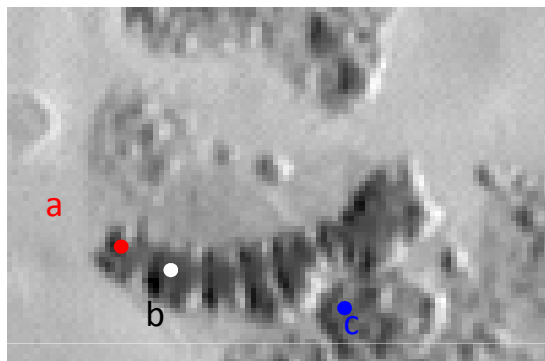

Na

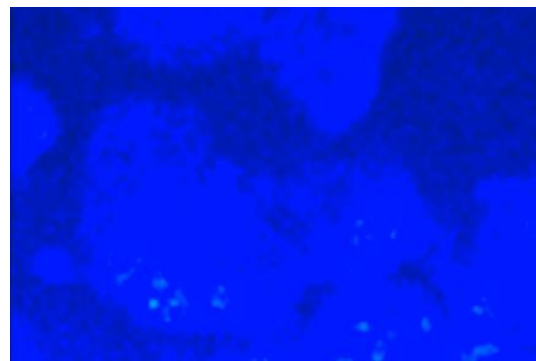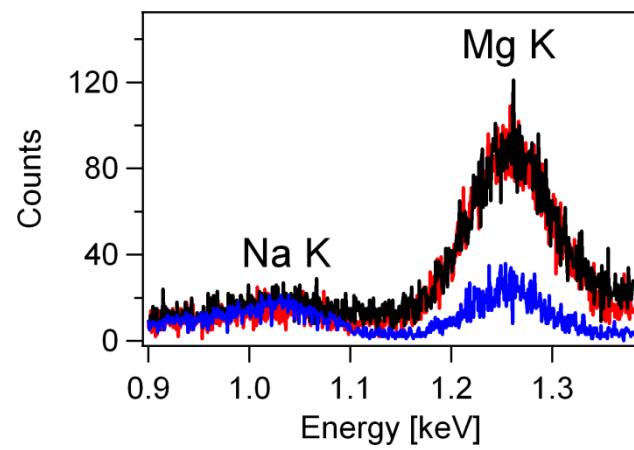

Supplement: Additional file 3 — Supporting information for Figure 4. Figures show phase contrast and Na distribution (XRF map) related to Figure 4. Spectra depict the low intensity signal from Na, compared to Mg (see XRF spectrum). See Figure 4 legend for technical details. [file 1743-8977-8-7-S3.PDF]
